# Dimensionality of the Spanish Version of the Universal Mental Health Literacy Scale Among Colombian Adults

**DOI:** 10.1155/nrp/5516224

**Published:** 2025-12-12

**Authors:** Adalberto Campo-Arias, Edwin Herazo, Jorge Mario Ortega-Iglesias

**Affiliations:** ^1^ Faculty of Health Sciences, Universidad del Magdalena, Santa Marta, Magdalena, Colombia, unimagdalena.edu.co; ^2^ General Directorate, Institute for Research on Human Behavior, Bogotá, Cundinamarca, Colombia

**Keywords:** adults, confirmatory factor analysis, dimensionality, mental health literacy, validation studies

## Abstract

The Universal Mental Health Literacy Scale for Adolescents (UMHL‐A) is a new 17‐item tool designed to assess adolescents’ mental health attitudes and knowledge. This scale can also be adapted for adults and is known as the UMHL. This study evaluated the dimensionality of the UMHL among Colombian adult high school students. A validation study involved 378 students aged 18–70 years (*M* = 20.06 and *SD* = 4.67), with 51.32% of the participants being female. The authors performed confirmatory factor analyses (CFAs) to compute loadings and goodness‐of‐fit statistics. Moreover, Kuder and Richardson’s Formula 20 was calculated for dichotomous items, and Cronbach’s alpha was calculated for Likert items to assess the internal consistency of each dimension. The CFA presented two subscales for the UMHL: attitudes and knowledge. Within the attitudes subscale, two dimensions were observed: help‐seeking and stigma/discrimination; this solution showed adequate goodness‐of‐fit indicators (X^*2*^ = 16.93, *df* = 14, *p* = 0.26, normalized X^*2*^ = 1.21, root mean square error of approximation (RMSEA) = 0.07 (90% confidence interval [90% CI]: 0.00–0.16), Comparative Fit Index (CFI) = 0.98, Tucker–Lewis Index (TLI) = 0.96, and normalized mean square residual (SRMR) = 0.05), while the knowledge subscale comprised a single dimension, similarly with acceptable goodness‐of‐fit coefficients (X^*2*^ = 26.08, *df* = 22, *p* = 0.25, normalized X^*2*^ = 1.19, RMSEA = 0.06 (90% CI: 0.00–0.14), CFI = 0.96, TLI = 0.94, and SRMR = 0.09). The internal consistency of help‐seeking dimension was appropriate (Cronbach’s alpha of 0.70), of stigma/discrimination dimension exhibited was low (Cronbach’s alpha of 0.45) and of knowledge dimension was acceptable (Kuder–Richardson’s Formula 20 of 0.68). In summary, the UMHL shows acceptable dimensionality; however, the stigma/discrimination aspect requires further examination. Additional studies are warranted.

## 1. Introduction

Mental health literacy encompasses beliefs and informal understandings related to mental health issues, including definitions of mental disorders, perceived causes, available treatments, and both formal and informal channels for seeking assistance [1–4].

Some perspectives contend that mental health literacy is an inadequately defined concept, necessitating further clarification [5]. However, various instruments exist to assess mental literacy in adolescents and adults; many of these scales are lengthy, and their reliability and validity remain questionable [6–8].

### 1.1. The Universal Mental Health Literacy Scale for Adolescents (UMHL‐A)

In 2023, Kågström et al. introduced the UMHL‐A, comprising 17 items and demonstrating acceptable dimensionality across two subscales. These subscales encompass two dimensions each: “attitudes” (which includes help‐seeking and stigma/discrimination) and “knowledge” (covering mental health and disorders). However, the authors omitted information on the internal consistency of the subscales and dimensions [9].

A study involving 268 Türkiyian participants aged 10–14 found that the UMHL‐A supported the initially suggested dimensionality, demonstrating satisfactory goodness‐of‐fit indicators, and the attitudes scale demonstrated strong internal consistency with a Cronbach’s alpha of 0.93. At the same time, the knowledge dimension reflected a comparable value of 0.86 [10]. Similarly, in a sample of 1208 Chinese students aged 11–15 years, researchers observed that the UMHL‐A’s dimensionality was satisfactory, and its subscales and dimensions presented acceptable internal consistency with Cronbach’s alpha values ranging from 0.71 to 0.82 [11]. Nevertheless, other analyses of the answer patterns of 223 participants, aged 10–14 years, from Türkiye, reported poor goodness‐of‐fit indicators. They dismissed the calculation of internal consistency due to the dichotomous nature of the response options for the knowledge subscale items [12]. However, internal consistency for scales with dichotomous response patterns can be reasonably calculated using the Kuder–Richardson coefficient [13]; this coefficient is equivalent to Cronbach’s alpha indicated for scales with items that offer ordinal or polytomous response patterns [14].

These findings are promising regarding the psychometric performance of the UMHL‐A [9–12]. However, these few studies cannot guarantee the dimensionality initially presented for the UMHL‐A. Furthermore, there is little information on the internal consistency of the instrument’s subscales and dimensions [15].

### 1.2. Practical Issues

The construction of a health measurement instrument typically begins with a concept or theory. This concept or theory must be easily reflected in the subscales, dimensions, and items initially endorsed by experts and subsequently confirmed by psychometric analysis and other empirical tests that demonstrate practical utility [15].

The UMHL‐A is an instrument based on Kutcher et al.’s (2016) conceptualization of mental health literacy: “Understanding how to obtain and maintain positive mental health, understanding mental disorders and their treatments, decreasing stigma related to mental disorders, and enhancing help‐seeking efficacy (knowing when and where to seek help and developing competencies designed to improve mental health care and self‐management capabilities).”

The studies cited in previous paragraphs are insufficient to guarantee the reliability (e.g., internal consistency) and validity (e.g., theoretical dimensionality) of the UMHL‐A [9–12]. Furthermore, it is worth noting that the reliability and validity coefficients of the instruments can vary significantly depending on the cultural and social backgrounds of the individuals who complete these tools [15]. These discrepancies may become more noticeable when this instrument is translated into a new language in which the linguistic equivalence of the first scale must be maintained, which frequently implies some modifications, preferably minor, in the wording of the items [16]. Consequently, it is imperative to evaluate the psychometric performance of the Spanish version of the UMHL‐A adapted for adults, which will be referred to as UMHL from now on [17].

Insufficient knowledge about mental health is widespread. For example, Kristina et al. [18] observed low scores for mental health literacy among university students in Yogyakarta. Mohammadi et al. [19] reported that 99% of the adults aged 18–90 in India had poor mental health literacy. This situation is concerning because limited mental literacy is one of the main factors contributing to the stigma and discrimination of mental disorders [20, 21]. Accurately and consistently exploring mental health literacy in diverse populations or samples is highly recommended to understand the scope of the problem and evaluate the effectiveness of efforts to increase knowledge about mental health [15].

### 1.3. The Current Study

This study examined the dimensionality of a Spanish version of the UMHL. It was decided to make this adaptation for adults because the tool comprises a few items [6–8] and the content of these items appeared to be suitable for adults as well [15]. Additionally, the authors assessed the internal consistency of the subscales and dimensions by utilizing the most suitable coefficients for the items’ response types: either Cronbach’s alpha (1951) or the Kuder–Richardson Formula 20 (1937). Additionally, according to the latest international guidelines for psychometric research [16], a second internal consistency coefficient was calculated: McDonald’s omega [22]. Two different coefficients with appropriate values for internal consistency provide greater assurance of the reliability of a health measurement scale, as they compensate for each other’s limitations and ensure the reliability of the measurement [23, 24]. The primary aim of the study was to assess the dimensionality of the UMHL in Colombian adult high school students.

## 2. Methods

### 2.1. Design and Participants

A cross‐sectional validation study was conducted to assess the dimensionality of the UMHL. Three‐hundred seventy‐eight students from 11 educational institutions in Santa Marta, Colombia, were included. A minimum sample size of approximately 400 participants is required for satisfactory convergence; most loadings are around 0.40, and three items are assigned to each dimension [25].

The participants ranged in age from 18 to 70 (*M* = 20.06 and *SD* = 4.67). Without exclusion criteria, only students working the night shift were included. In Colombia, night shift schools are offered for teenagers aged 16 and older. Table 1 provides additional demographic information.

**Table 1:** Demographic characteristics of participants.

Variable	Frequency	%
Gender		
** ** *Male*	180	47.62
** ** *Female*	194	51.32
** ** *Nonbinary*	4	1.06
Grade		
** ** *Sixth*	7	1.85
** ** *Seventh*	12	3.17
** ** *Eighth*	2	0.53
** ** *Ninth*	47	12.44
** ** *Tenth*	8	2.12
** ** *Eleventh*	302	79.89
Family income		
** ** *Low*	297	78.57
** ** *Middle*	75	19.84
** ** *High*	6	1.59
Marital status		
** ** *Single*	259	68.52
** ** *Free union*	76	20.11
** ** *Married*	31	8.19
** ** *Separated*	8	2.12
** ** *Widowed*	4	1.06
Employee		
** ** *Yes*	180	47.62
** ** *No*	198	52.38
Religious affiliation		
** ** *Catholic*	100	26.46
** ** *Non-Catholic Christian*	132	34.92
** ** *None*	116	30.68
** ** *Muslim*	8	2.12
** ** *Mormon*	2	0.53
** ** *Other*	20	5.29

The sample exhibited a balanced gender distribution, with a slight majority (51.32%) identifying as female. Most participants (79.89%) were enrolled in the 11th grade. Regarding family income, 78.57% of the participants reported belonging to low‐income households. Additionally, 61.38% of the participants identified as Christian, encompassing Catholic and non‐Catholic affiliations.

### 2.2. Instrument

The UMHL comprises 17 items divided into two subscales: attitudes and knowledge. The attitudes subscale features two dimensions: help‐seeking (Items 1, 2, 4, 6, and 7) and the stigma/discrimination complex (Items 3, 5, and 8). Meanwhile, the knowledge subscale encompasses two dimensions: knowledge of mental health (Items 9 and 14–17) and understanding of mental disorders (Items 10–12 and 13) [9].

Responses for the attitude’s subscale are on a five‐point scale, ranging from “*completely disagree*” (coded as 1) to “*completely agree*” (coded as 5). The knowledge subscale presents a simple dichotomous choice: yes (coded as 1) and no (coded as 0). A higher score indicates a more favorable attitude toward mental health (less stigma/discrimination) and greater knowledge of mental health issues and disorders [9].

### 2.3. Procedure

#### 2.3.1. UMHL’s Translation and Back‐Translation

Two bilingual mental health professionals (the first authors) independently translated the UMHL from English to Spanish. The Spanish versions were checked and confirmed to match since the English text uses straightforward sentences that are easy to translate. It is crafted with common expressions, avoiding negative phrasing and derogatory adjectives that could confuse Spanish speakers.

Finally, an independent professional Spanish English translator performed a new back‐translation into English. This back‐translation mirrored the original English version and was accepted with minor corrections. The last modification to better suit adults involved altering item 2 from [′peers′] to “study or work partners” and item 6 from [′adults′] to “romantic partners”. Moreover, the UMHL was adjusted by removing the option “I do not know,” as this choice tends to bias responses from Spanish‐speaking participants for linguistic or cultural factors [16]. Finally, the clarity of the items was assessed qualitatively with a group of 10 students. During the application of the final questionnaire, no student asked about the meaning or significance of the items. The original English version and the Spanish adaptation for adults are included in the Supporting material.

#### 2.3.2. UMHL Application

Students participated in a group effort to complete a paper booklet that collected demographic details and featured the Spanish version of the UMHL. This process took place in the designated areas of each educational institution. Data collection occurred from the first week of September to the first week of November 2024.

### 2.4. Statistical Analysis

To demonstrate the dimensional nature of the UMHL, the authors performed confirmatory factor analyses (CFAs) on the subscales proposed by Kågström et al. [9]. The loadings were evaluated, and the following goodness‐of‐fit statistics were computed: chi‐square, normalized chi‐square, root mean square error of approximation (RMSEA) with a 90% confidence interval (90% CI), Comparative Fit Index (CFI), Tucker–Lewis Index (TLI), and normalized mean square residual (SRMR). The authors would consider the dimensionality of the UMHL acceptable if the normalized chi‐square is below 3.00 [26], the RMSEA is under 0.06, and both the CFI and TLI exceed 0.90. Additionally, the SRMR should be below 0.05 [27].

Internal consistency was assessed using Kuder and Richardson’s test (1937) for dichotomous items and the equivalent of Cronbach’s alpha (1951) for polytomous ones. Additionally, McDonald’s omega (1970) was calculated. Omega serves as a more accurate measure of consistency, especially when the “tau‐equivalent model” assumption is not met, which presumes that every item in the scale measures the same latent trait essential for Cronbach’s alpha calculations [24]. Generally, consensus values range from 0.70 to 0.95 [28]. However, values of 0.60 are acceptable if the upper limit of the CI is 0.70 or when developing new instruments [29, 30]. The statistical coefficients were computed using STATA Version 17.0 and Jamovi Version 2.3.

### 2.5. Ethical Issues

The Research Ethics Committee of the Universidad del Magdalena approved this project (Minutes 006 from the ordinary June 20, 2024, session). The parents of the students signed their informed consent, and students consented for participation in the study. The questionnaire was designed to be anonymous, omitting names and surnames to ensure confidential responses. The research tools utilized were freely accessible for academic use or obtained with the authors’ permission. This approach aligns with national and international guidelines for research involving human participants [31].

## 3. Results

The score on the help‐seeking dimension was observed between 5 and 25 (*M* = 18.48 and *SD* = 3.55); the stigma/discrimination dimension, 3 and 15 (*M* = 12.00 and *SD* = 1.88); and the attitudes’ subscale, 13 and 40 (*M* = 30.48 and *SD* = 4.70). Loadings were between 0.39 and 0.65. Only Item 3 (getting along with others is important for mental health) was inferior to 0.40. All loadings of the attitudes subscale are detailed in Table 2.

**Table 2:** Factor loadings for the attitudes subscale.

Item	Factor	
	1	2
1. Talking about my feelings with someone helps to improve my mental health	0.60	
2. I am comfortable talking to my study or work partners about my feelings	0.50	
3. Getting along with others is important for mental health		0.39
4. If I experienced mental problems, I would seek help	0.65	
5. If someone I care about had mental health problems for a long time, I would recommend them to get professional help		0.59
6. I am comfortable talking to a romantic partner about my feelings	0.52	
7. If I had a mental disorder, I would speak about it with others	0.57	
8. I would be willing to continue a friendship with someone who developed a mental health problem		0.47

The parsimonious model showed limited goodness‐of‐fit coefficients: X^2^ = 46.65, *df* = 19, *p* < 0.001, normalized X^2^ = 2.46, RMSEA = 0.17 (90% CI: 0.11–0.23), CFI = 0.82, TLI = 0.73, and SRMR = 0.08. However, with the inclusion of the covariances of the item pairs 2 ∗ 7, 3 ∗ 4, 3 ∗ 6, 5 ∗ 6, and 6 ∗ 8, the model fit adequately: X^*2*^ = 16.93, *df* = 14, *p* = 0.26, normalized X^*2*^ = 1.21, RMSEA = 0.07 (90% CI: 0.00–0.16), CFI = 0.98 TLI = 0.96, and SRMR = 0.05.

The scores on the mental health dimension ranged from 0 to 5 (*M* = 2.49 and *SD* = 1.56); the mental disorders dimension, 0 to 4 (*M* = 3.67 and *SD* = 0.77); and the knowledge subscale, 0 to 9 (*M* = 6.16 and *SD* = 1.92). Loadings were between 0.28 and 0.66. Four items (9 and 15–17) showed above 0.40. All loadings of the knowledge subscale are presented in Table 3.

**Table 3:** Factor loadings for the knowledge subscale.

Item	Loading
9. How I get along with others affects my mental health	0.41
10. Mental illnesses are caused by different things	0.35
11. Mental health impacts people’s behavior	0.37
12. Mental health affects people’s emotions	0.30
13. Depression is one of the most common mental illnesses among young people	0.35
14. The way people feel over time is a sign of their mental health	0.28
15. How people think about things affects their mental health	0.56
16. How people get along with others affects how they feel	0.65
17. How people think about things affects how they feel	0.66

The two‐dimensional solution of the knowledge subscale did not reach convergence; therefore, the one‐dimensional solution was chosen. The parsimonious one‐dimensional model showed a poor goodness‐of‐fit: X^*2*^ = 63.28, *df* = 27, *p* < 0.001, normalized X^*2*^ = 2.34, RMSEA = 0.16 (90% CI: 0.11–0.22), CFI = 0.65, TLI = 0.54, and SRMR = 0.12. Nevertheless, when the covariances of the item pairs 10 ∗ 12, 10 ∗ 16, 11 ∗ 12, 12 ∗ 13, and 16 ∗ 17 were added to the model, the model fit acceptably: X^*2*^ = 26.08, *df* = 22, *p* = 0.25, normalized X^*2*^ = 1.19, RMSEA = 0.06 (90% CI: 0.00–0.14), CFI = 0.96, TLI = 0.94, and SRMR = 0.09.

The internal consistencies of dimensions and subscales were between 0.45 and 0.74. Only the stigma/discrimination dimension presented values below 0.50. All internal consistency coefficients are presented in Table 4.

**Table 4:** Internal consistency of subscales and dimensions.

Dimension or subscale	*Alpha*	*Omega*
Help‐seeking	0.70	0.71
Stigma/discrimination	0.45	0.48
Attitudes	0.73	0.74
Mental health	0.66^∗^	0.67
Mental disorders	0.67^∗^	0.67
Knowledge	0.68^∗^	0.70

*Note*: ^∗^Kuder–Richardson coefficient for dichotomous scales.

## 4. Discussion

The current analyses corroborate the two‐dimensional subscales of the Spanish version of the UMHL, which was adapted for adults. Nonetheless, the knowledge subscale presents the best goodness‐of‐fit coefficients as a one‐dimensional structure in a sample of night‐shift high school students in Colombia.

The authors of the present study observed that the two‐dimensional solution fits the data of the UMHL attitudes subscale, while the one‐dimensional solution fits the data of the knowledge subscale. Nevertheless, Kågström et al. [9], Ciydem and Avci [10], and Wang et al. [11] reported that the UMHL‐A consists of two subscales, making it a bidimensional scale. In contrast, Uysal et al. [12] noted that the data did not align with the dimensionality suggested when the UMHL‐A was presented. These discrepancies underscore the necessity for cultural and contextual adaptation of the instrument, as variations in populations and settings can impact dimensionality [16].

While the attitudes subscale exhibits a robust structure, Item 3 requires conceptual revision or adjustment. Initial modeling yielded inadequate fit indices. However, incorporating covariances between specific item pairs significantly improved the model fit, resulting in a parsimonious model with optimal fit. This finding suggests that content overlaps or thematic similarities between certain items contribute to their intercorrelation [15]. Additionally, a further review of the items in the stigma/discrimination subscale raises questions about their content validity. Item 3 seems more related to knowledge than to stigma and discrimination. Item 5 might be better classified in the help‐seeking subscale. Only Item 8 aligns with the traditional view of the stigma/discrimination complex in mental health [32].

The knowledge subscale’s unidimensional solution did not provide an adequate fit, highlighting the need to consider correlations between items that share content or overlapping conceptual characteristics. The substantial improvement in fit after incorporating covariances suggests that the instrument’s structure may require further refinement to reflect better the relationships between the items and the measured construct [15, 33].

Typically, the suggested or measured dimensionality is deemed acceptable when three out of the five most commonly reported goodness‐of‐fit indicators meet the recommended criteria [27]. Given the minimal factor loading (wording or content), Item 14 of the UMHL should be revised for future studies since it undermines the subscale’s dimensionality (and internal consistency) and the “knowledge of mental disorders” dimension. Cultural factors likely play a crucial role in the poor performance of this item since it explores behavior over time, rather than at the present moment [16]. Scales need adjustments based on empirical findings [15, 33].

This analysis revealed varying internal consistency among the subscales and dimensions. The stigma/discrimination dimension was significantly lower than the generally recommended threshold. This result challenges earlier research that indicated high internal consistency values for the UMHL‐A subscales and dimensions [10, 11].

Internal consistency values between 0.70 and 0.95 are commonly recognized [28]. However, a value of 0.60 is permissible if the upper limit of the CI is 0.70 or in the context of developing new instruments [29, 30]. These findings emphasize the importance of context‐specific validation processes, particularly when applying instruments to diverse populations [16]. Instruments with good reliability and validity indicators are usually more helpful in clinical practice and applied research [33]. Future research should consider implementing exploratory and CFAs in larger, more diverse samples to refine the instrument further and improve its psychometric properties [25].

### 4.1. Practical Considerations

Generally, psychometrists speak of the “psychometric properties” of health measurement instruments. However, the term is somewhat imprecise or inappropriate, given that the findings after applying one of these measurement scales in a population show more of the participants’ particular response pattern than the instrument’s intrinsic properties [33]. Consequently, divergent performances will probably reflect the disparities in the populations more than in the instrument itself, as they are always latent variables [15, 33].

These findings are promising as they are a step forward in measuring mental health literacy on a brief and practical scale. Theoretically, in psychometrics, it is expected that these response patterns in different populations are similar enough to have a measurement scale that allows comparisons between groups with various cultural and social characteristics [33]. Psychometric coefficients should always be understood as an inferential approximation [15]. In the present study, the low loading of some items and the poor internal consistency of the stigma/discrimination dimension necessitate that these items be revised, at least in adults and Spanish. It is evident that the UMHL is still under construction and requires further validation of its dimensionality and internal consistency across different populations worldwide [17].

### 4.2. Strengths and Limitations of the Study

The present study has the strength of reporting for the first time the dimensionality of the UMHL in a sample of adults and shows that the instrument can be used not only in adolescents. Furthermore, this is the first study to show that a Spanish version of the UMHL retains linguistic equivalence with the English version. Adapted Item 2 (I am comfortable talking to my study or work partners about my feelings) showed an acceptable loading of 0.50, and Item 6 (I am comfortable talking to a romantic partner about my feelings) reached a loading of 0.52. To date, detailed indicators related to the psychometric performance of the UMHL‐A among early adolescents in China and Türkiye have been documented [10–12].

Moreover, the current research calculated two internal consistency measures to determine the reliability of the UMHL. However, the study includes only adults who attend school, work at night, and are of low income. Future evaluations of the psychometric performance of the UMHL should be extended to samples from diverse backgrounds. It must pay attention to the wording change in Items 2 and 6 because it relates to people or situations that may vary with the participants’ age [16].

## 5. Conclusions

The authors conclude that the UMHL attitudes subscale exhibits a bidimensional structure, whereas the knowledge subscale remains unidimensional for Colombian adult night‐shift high school students. The low internal consistency of the stigma/discrimination dimension highlights the need to revise its wording or create new items. These results should be viewed as preliminary. Additional research is needed to validate the dimensionality of the UMHL.

## Conflicts of Interest

The authors declare no conflicts of interest.

## Author Contributions

**Adalberto Campo-Arias** was involved in the conception and design of the article, conducted the statistical analysis, interpreted and discussed the results, wrote the draft, and approved the final version. Email: acampoa@unimagdalena.edu.co **Edwin Herazo** contributed to the conception and design of the study, the interpretation and discussion of the results, the critical review, and the endorsement of the final version. Email: eh@comportamientohumano.org **Jorge Mario Ortega-Iglesias** participated in analyzing and interpreting the data, discussed the findings, and endorsed the final draft. Email: jortegai@unimagdalena.edu.co.

## Funding

The Vice‐Rectorate for Research of the Universidad del Magdalena, Santa Marta, in collaboration with the Instituto de Investigación del Comportamiento Humano, Bogotá, Colombia, supported the project’s funding (VIN2024207 Resolution).

## Supporting Information

Supporting Materials: Adapted Spanish version of the Universal Mental Health Literacy Scale adapted for adults.

## Supporting information


**Supporting Information** Additional supporting information can be found online in the Supporting Information section.

## Data Availability

Data used to support the findings of this study are available on request from the corresponding author.
