# Supplementary material for: Dimensionality of the Spanish Version of the Universal Mental Health Literacy Scale Among Colombian Adults
Source: Nurs Res Pract. 2025 Dec 12;2025:5516224. doi: 10.1155/nrp/5516224 (PMC12767458; doi:10.1155/nrp/5516224)
Supplement: Supplementary file 1 — Supporting Information Additional supporting information can be found online in the Supporting Information section. [file NRP-2025-5516224-s001.docx]

**Supplementary material**

**Adapted Spanish version of the Universal Mental Health Literacy Scale adapted for adults**

1. Talking about my feelings with someone helps to improve my mental health [*Hablar de mis sentimientos con alguien ayuda a mejorar mi salud mental*].
2. I am comfortable talking to my peers about my feelings [*Es cómodo hablar de mis sentimientos con mis compañeros (as) de estudio o trabajo*].
3. Getting along with others is important for mental health [*Llevarse bien con las demás personas es importante para la salud mental*].
4. If I experienced mental problems, I would seek help [*Si yo tuviera problemas mentales, buscaría ayuda*].
5. If someone I care about had mental health problems for a long time, I would recommend them to get professional help [*Si una persona importante para mí tuviera problemas de salud mental durante mucho tiempo, le recomendaría que busque ayuda profesional*].
6. I am comfortable talking to adults about my feelings [*Es cómodo hablar de mis sentimientos con mi pareja sentimental*].
7. If I had a mental disorder, I would speak about it with others [*Si tuviera un trastorno mental, hablaría de ello con otras personas*].
8. I would be willing to continue a friendship with someone who developed a mental health problem [*Estaría dispuesto a continuar una amistad con alguien que presentó un problema de salud mental*].
9. How I get along with others affects my mental health [*La forma en que me llevo con las demás personas afecta mi salud mental*].
10. Mental illnesses are caused by different things [*Los trastornos mentales tienen diferentes causas*].
11. Mental health impacts people’s behaviour [*La salud mental se refleja en el comportamiento de las personas*].
12. Mental health affects people’s emotions [*La salud mental afecta la expresión de las emociones de las personas*].
13. Depression is one of the most common mental illnesses among young people [*La depresión es uno de los trastornos mentales más comunes en la actualidad*].
14. The way people feel over time is a sign of their mental health [*La forma en que las personas se comportan a lo largo del tiempo es una señal de su salud mental*].
15. How people think about things affects their mental health [*La forma en que la gente piensa sobre las cosas que le pasan afecta su salud mental*].
16. How people get along with others affects how they feel [*La forma en que las personas se llevan con las demás personas afecta cómo se sienten*].
17. How people think about things affects how they feel) [*La forma en que las personas piensan sobre las cosas que le suceden afecta cómo se sienten*].
